# Redox Bridling of GIRK Channel Activity

**DOI:** 10.1093/function/zqad027

**Published:** 2023-06-07

**Authors:** Anna Boccaccio, Rocio K Finol-Urdaneta

**Affiliations:** Institute of Biophysics, National Research Council, Via De Marini 6, 16149 Genoa, Italy; Faculty of Science, Medicine and Health, University of Wollongong, Wollongong, NSW 2522, Australia

## **A Perspective on** “Oxidation Driven Reversal of PIP2-dependent Gating in GIRK2 Channels”

G protein-gated inwardly rectifying potassium (GIRK, Kir3.x) channels belong to the large family of inwardly rectifying potassium (Kir) channels expressed throughout the body. Activation and consequent opening of GIRK channels allow inward flow of potassium (K^+^) ions into the cell resulting in membrane potential hyperpolarization and decreased excitability. Thus, GIRK channels play a key role in regulating the activity of neurons and controlling important physiological processes including neuronal excitability, heart rate, and pain perception.^1^

GIRK channels are integral membrane proteins, existing as homo- or heterotetramers. Each monomer features two membrane-spanning helices (M1 and M2), a re-entrant P-loop for controlling ion permeation and selectivity, and extensive intracellular amino- and carboxy-termini crucial for channel gating. Permeation is regulated by an inner helix gate formed by the M2 segments and a cytoplasmic G-loop gate.^1^

Activation of GIRK channels is mediated by the direct interaction of Gβγ subunits, released from various G protein-coupled receptors (GPCRs) upon the activation of inhibitory neurotransmitter receptors. However, the activity of GIRK channels depends on the presence of the membrane anionic phospholipid phosphatidylinositol-4,5-bisphosphate (PI(4,5)P_2_ or PIP_2_) while it is also modulated by ubiquitously present sodium (Na^+^) ions. Furthermore, GIRK channels are too regulated by cholesterol, phosphorylation, ethanol, etcetera.^1^ The crystal structures of recombinant GIRK channels have offered valuable insights into how they are functionally regulated by various ligands. Thus, channel opening is facilitated by PIP_2_ at the plasma membrane, whereas Gβγ and Na^+^ modulate the channel’s interaction with PIP_2_ through conformational changes that govern the gating process.^2^

The intracellular milieu is a reducing environment characterized by a balanced redox state. This state is crucial to support cellular processes while serving as a protective shield against damaging reactive oxygen species (ROS) thereby facilitating enzymatic reactions and energy production.^3^ GIRK channels exhibit low basal activity in reducing intracellular environments. Nevertheless, the channel's behavior in native tissues is influenced by a variety of cellular factors, which complicate the interpretation of their specific contributions to channel gating.

In a recent *Function* publication, Lee et al. effectively bypassed the complexity of cellular systems to study the regulation of GIRK channels.^4^ The team employed an acellular *in vitro* flux assay to manipulate the redox state of the functional channel protein within liposomes made of determined phospholipid mixtures. Subsequently, they assessed GIRK2-mediated fluxes upon systematic replacement of key modulatory co-factors. This method successfully reproduced the channel’s typical behavior (ie, low basal activity in reducing conditions) while also uncovering an oxidation-dependent anomalous enhancement of GIRK2 activation.

It was observed that in the absence of stringent redox control, as is commonly provided by healthy cells, the GIRK2 protein undergoes oxidation at two crucial cysteine residues during purification. These cysteines have different susceptibility to environmental oxidation, and their oxidized state appears to play a pivotal role in GIRK2 gating. Two distinct effects were observed upon oxidation: (i) the loss of PIP_2_ and Na^+^-dependent activity, linked to cysteine 65 and (ii) a slower rise in basal activity associated with cysteine 190.

The N-terminal cysteine at position 65 of GIRK2 is highly conserved in the Kir channel family and it is strategically located adjacent to lysine 64, which contributes to the PIP_2_ binding site. Moreover, C65 is positioned toward a neighboring subunit’s pocket, which is involved in the Na^+^ binding site (Figures 5B and 7A in Lee et al.^4^). Notably, C65 plays a role in GIRK current inhibition by Cd^2+^ through its direct interaction with a high-affinity locus at the entrance of the Na^+^ binding site.^5^

Cysteine 190, a conserved residue among the Kir3 and Kir6 subfamilies, is positioned at the cytoplasmic end of the TM2 inner helix near the gate residue phenylalanine 192. It faces outward, toward the outer helix TM1, lipid membrane, and the phosphatidylinositol molecule tail. Unlike the cytoplasmically exposed C65, C190 appears buried within the channel protein consistent with the slower time course of oxidation revealed by the study.

Lee et al.^4^ uncover how the protein purification environment, especially harsh oxidizing conditions, affects GIRK2 channel function. They examined different GIRK2 structures with PIP_2_, Na^+^, and Gβγ, emphasizing slight rearrangements near the neighboring regions of C65 and C190. This structural comparison provides evidence that modifications to these cysteines may impact the channel’s gating mechanism. The conservation of specific oxidizable cysteine residues across multiple species in GIRK channels suggests that oxidation-mediated regulation may influence their physiological function in various organisms.

Recombinant GIRK channels expressed in *Xenopus laevis* oocytes have been shown to display redox-dependent gating mediated through cytoplasmic cysteine residues.^6–8^ However, experiments conducted on homo- and heteromeric GIRK channels in cellular systems have yielded conflicting results. Specifically, GIRK channels exhibited increased activity upon application of ROS^8^ and the reducing agent DTT.^6^ Given the apparent impact of protein oxidation on GIRK channel function, investigating the potential influence of lipid peroxidation on membrane/liposome composition and GIRK channel behavior could yield valuable insight.

When there is an imbalance between ROS and antioxidants, free radicals generated during cell metabolism trigger oxidative stress. This stress damages proteins, lipids, and DNA, disrupting cellular homeostasis. Hence, it is implicated in various physiological and pathophysiological processes. Mounting evidence has implicated GIRK channel dysfunction in the pathophysiology of neurological and cardiovascular disorders.^9,10^ However, the precise mechanisms by which GIRK channels are involved in these diseases remain incompletely understood.

The recent findings from the Nichols’ lab, combined with previous research on cellular systems, highlight the oxidative state as a crucial factor influencing GIRK function. This could hold significant relevance in scenarios where heightened metabolic activity, such as during episodes of neuronal firing and rapid cardiac myocyte contractions, overlaps with pathological conditions characterized by increased production of ROS or impaired antioxidant capacity. Considering the influence of ROS on the activity of ion channels and transporters, unbridled activation of GIRK channels may serve as a critical link connecting cellular metabolic state, ionic homeostasis, and excitability.

## Data Availability

This article does not include data.
